# Estimation and testing for the effect of a genetic pathway on a disease outcome using logistic kernel machine regression via logistic mixed models

**DOI:** 10.1186/1471-2105-9-292

**Published:** 2008-06-24

**Authors:** Dawei Liu, Debashis Ghosh, Xihong Lin

**Affiliations:** 1 Center for Statistical Sciences, Brown University, Providence, RI 02912, USA; 2 Departments of Statistics and Public Health Sciences, Pennsylvania State University, University Park, PA 16802, USA; 3 Department of Biostatistics, Harvard School of Public Health, 655 Huntington Avenue, Boston, MA 02115, USA

## Abstract

**Background:**

Growing interest on biological pathways has called for new statistical methods for modeling and testing a genetic pathway effect on a health outcome. The fact that genes within a pathway tend to interact with each other and relate to the outcome in a complicated way makes nonparametric methods more desirable. The kernel machine method provides a convenient, powerful and unified method for multi-dimensional parametric and nonparametric modeling of the pathway effect.

**Results:**

In this paper we propose a logistic kernel machine regression model for binary outcomes. This model relates the disease risk to covariates parametrically, and to genes within a genetic pathway parametrically or nonparametrically using kernel machines. The nonparametric genetic pathway effect allows for possible interactions among the genes within the same pathway and a complicated relationship of the genetic pathway and the outcome. We show that kernel machine estimation of the model components can be formulated using a logistic mixed model. Estimation hence can proceed within a mixed model framework using standard statistical software. A score test based on a Gaussian process approximation is developed to test for the genetic pathway effect. The methods are illustrated using a prostate cancer data set and evaluated using simulations. An extension to continuous and discrete outcomes using generalized kernel machine models and its connection with generalized linear mixed models is discussed.

**Conclusion:**

Logistic kernel machine regression and its extension generalized kernel machine regression provide a novel and flexible statistical tool for modeling pathway effects on discrete and continuous outcomes. Their close connection to mixed models and attractive performance make them have promising wide applications in bioinformatics and other biomedical areas.

## Background

The rapid progress in gene expression array technology in the past decade has greatly facilitated our understanding of the genetic aspect of various diseases. Knowledge-based approaches, such as gene set or pathway analysis, have become increasingly popular. In such gene sets/pathways, groups of genes act in concert to accomplish tasks related to a cellular process and the resulting genetic pathway effects may manifest themselves through phenotypic changes, such as occurrence of disease. Thus it is potentially more meaningful to study the overall effect of a group of genes rather than a single gene, as single-gene analysis may miss important effects on pathways and difficult to reproduce from studies to studies [1]. Researchers have made significant progress in identifying metabolic or signaling pathways based on expression array data [2,3]. Meanwhile, new tools for identification of pathways, such as GenMAPP [4], Pathway Processor [5], MAPPFinder [6], have made pathway data more widely available. However, It is a challenging task to model the pathway data and test for a potentially complex pathway effect on a disease outcome.

One way to model pathway data is through the linear model approach, where the pathway effect is represented by a linear combination of individual gene effects. This approach has several limitations. Activities of genes within a pathway are often complicated, thus a linear model is often insufficient to capture the relationship between these genes. Furthermore, genes within a pathway tend to interact with each other. Such interactions are not taken into account by the linear model approach.

In this paper we propose a nonparametric approach, the kernel machine regression, to model a pathway effect. The kernel machine method, with the support vector machine (SVM) as a most popular example, has emerged in the last decade as a powerful machine learning technique in high-dimensional settings [7,8]. This method provides a flexible way to model linear and nonlinear effects of variables and gene-gene interactions, unifies the model building procedure in both one- and multi-dimensional settings, and shows attractive performance compared to other nonparametric methods such as splines.

Liu et al. [9] proposed a kernel machine-based regression model for continuous outcomes. In this paper, we propose a logistic kernel machine regression model for binary outcomes, where covariate effects are modeled parametrically and the genetic pathway effect is modeled parametrically or nonparametrically using the kernel machine method. A main contribution of this paper is to establish a connection between logistic kernel machine regression and the logistic mixed model. We show that the kernel machine estimator of the genetic pathway effect can be obtained from the estimator of the random effects in the corresponding logistic mixed model. This connection provides a convenient vehicle to connect the powerful kernel machine method with the popular mixed model method in the statistical literature. This mixed model connection also provides an unified framework for statistical inference for model parameters, including the regression coefficients, the nonparametric genetic pathway function, and the regularization and kernel parameters. Based on the proposed logistic kernel machine regression model, we develop a new test for the nonlinear pathway effect on disease risk. An appealing feature of the proposed test is that it performs well without the need to correctly specify the functional form of the effects of each gene or their interactions. This feature has a significant practical implication when analyzing genetic pathway data, as the true relationship between the pathway and the disease outcome is often unknown. We extend the results to generalized kernel machine regression for a class of continuous and discrete outcomes and discuss its connection with generalized linear mixed models [10].

Recently, Wei and Li [11] proposed a nonparametric pathway-based regression (NPR) to model pathway data. NPR is a pathway-based gradient boosting procedure, where the base learner is usually a regression or classification tree. It provides a flexible approach in modeling pathways and interactions among genes within a pathway. Michalowski et al. [12] proposed a Bayesian Belief Network approach for pathway data. Neither method is likelihood-based. Thus parameter estimation and inference cannot be casted within a unified likelihood framework. It is hence difficult to estimate and quantify the overall pathway effect on disease risk and assess its statistical uncertainty. Secondly, a primary interest in this paper is to test for the statistical significance of the overall pathway effect on the risk of a disease. Both NPR and Bayesian belief network do not provide such a statistical test for the pathway effect. For example, NPR uses an importance score to rank the relative importance of each pathway. It lacks formal inferential procedure for assessing the statistical significance of a pathway. Further, when considering a single pathway, the importance score loses its meaning in assessing the importance of a pathway. Our method, on the other hand, is based on penalized likelihood, and estimation and inference can be conducted in a systematic manner within the likelihood framework. We also propose a formal statistical test for the significance of a pathway effect on the risk of a disease.

Goeman et al. [13] proposed a linear mixed model to relate the pathway effect with a continuous outcome. They modeled the pathway effect using a linear function with each gene entering into the model as a regressor. They assumed the regression coefficients of the gene as random from a common distribution with mean 0 and an unknown variance. The pathway effect can then be tested through a variance component test for random effects. Our approach is different from theirs in the following aspects. First, we model the pathway effect by allowing for a nonparametric model rather than a parametric one. As we commented earlier, the highly complicated nature of activities of genes within a pathway makes the linear model assumption untenable. Secondly, the kernel function used in kernel machine regression usually contains unknown tuning parameters. The parameter is present under the alternative hypothesis but disappears under null hypothesis. This makes tests as proposed in [13,14] not applicable. Our proposed test, on the other hand, works quite well under this scenario. Third, Goeman et al. [14] extended their linear model results to discrete outcomes using basis functions. A key advantage of the kernel machine approach over this basis approach for modeling multi-gene effects is that one does not need to specify bases explicitly, which is often difficult for high-dimensional data especially when interactions are modeled.

## Results

### Analysis of prostate cancer data

In this section, we apply the proposed logistic kernel machine regression model (3) as described in the Methods section to the analysis of a prostate cancer data set. The data came from the Michigan prostate cancer study [15]. This study involved 81 patients with 22 diagnosed as non-cancerous and 59 diagnosed with local or advanced prostate cancer. Besides the clinical and demographic covariates such as age, cDNA microarray gene expressions were also available for each patient. The early results of Dhanasekaran et al. [15] indicate that certain functional genetic pathways seemed dys-regulated in prostate cancer relative to non-cancerous tissues. We are interested in studying how a genetic pathway is related to the prostate cancer risk, controlling for the covariates. We focus in this analysis on the cell growth pathway, which contains 5 genes. The pathway we describe was annotated by the investigator (A. Chinnaiyan) and is simply used to illustrate the methodology. Of course, one could take the pathways stored in commercial databases such as Ingenuity Pathway Analysis (IPA) and use the proposed methodology based on those gene sets.

The outcome was the binary prostate cancer status and the covariate includes age. Since the functional relationship between the cell growth pathway and the prostate cancer risk is unknown, the kernel machine method provides a convenient and flexible framework for the evaluation of the pathway effect on the prostate cancer risk. Specifically, we consider the following semiparametric logistic model

(1) logit(*P*(*y *= 1)) = *β*_0 _+ *β*_1_age + *h*(gene_1_, ..., gene_5_),

where *h*(·) is a nonparametric function of 5 genes within the cell growth pathway. The detail of the estimation procedure is provided in the Methods section. In summary, we fit this model using the kernel machine method via the logistic mixed model representation and using the Gaussian kernel function in estimating *h*(·). Under the mixed model representation, we estimated (*β*_0_, *β*_1_) and *h*(·) using penalized quasi-likelihood (PQL), and estimated the smoothing parameter *τ *and the Gaussian kernel scale parameter *ρ *simultaneously by treating them as variance components. The results are presented in Table 1.

**Table 1:** Analysis of prostate cancer data.

Covariate	Estimate	S.E.	P-value
Intercept	0.9893	2.7552	0.7205
Age	-0.0140	0.0425	0.7430
*τ*	4.7362	3.6190	
*ρ*	1.9093	0.6603	

Score test for the genetic pathway effect *H*_0_: *h*(***z***) = 0:			

Test	P-value		
KM	< 0.0001		
GT	0.0661		

Parameter estimates and score test of the logistic kernel machine regression model for the genetic pathway effect applied to the prostate cancer data. In the table, KM stands for Kernel machine method using the Gaussian kernel, and GT for global test of Geoman et al. [13] assuming linearity.

The test for the cell growth pathway effect on the prostate cancer status *H*_0_: *h*(***z***) = 0 vs *H*_1_:*h*(***z***) ≠ 0 was conducted using the proposed score test as described in the Methods section. For the purpose of comparison, we also conducted the global test proposed by Goeman et al. [13] that assumed a linear pathway effect. Note that our test allows a nonlinear pathway effect and gene-gene interactions. Table 1 gives the p-values for both tests. The p-value of our test suggests that cell growth pathway has a highly significant effect on the disease status, while the test from Goeman et al. [13] indicates only marginal significance of the growth pathway effect.

### Simulation Study for the Parameter Estimates

We conducted a simulation study to evaluate the performance of the parameter estimates of the proposed logistic kernel machine regression by using the logistic mixed model formulation. We considered the following model

(2) logit(*P*(*y*_*i *_= 1)) = *x*_*i *_+ *h*(*z*_*i*1_, ⋯, *z*_*ip*_),

where the true regression coefficient *β *= 1. We consider *p *= 5 and set *h*(*z*_1_, ..., *z*_5_) = 2{sin(*z*_1_) – $z_{2}^{2}$ + *z*_1 _exp(-*z*_3_) -sin(*z*_2_) cos(*z*_3_) + $z_{4}^{2}$ + sin(*z*_4_) cos(*z*_1_) + $z_{5}^{2}$ + *z*_3_*z*_5_}. To allow *x*_*i *_and (*z*_*i*1_, ⋯, *z*_*ip*_) to be correlated, *x*_*i *_was generated as *x*_*i *_= sin(*z*_*i*1_) + 2*u*_*i*_, where *u*_*i *_and *z*_*ij *_(*j *= 1, ⋯, *p*) follow independent Uniform(-0.5, 0.5). The Gaussian kernel was used throughout the simulation. All simulations ran 300 times. Settings 1, 2, and 3 correspond to sample size *n *= 100, 200, and 300, respectively.

The simulation results are shown in Table 2. Due to the multi-dimensional nature of the variables ***z***, it is difficult to visualize the fitted curve $\hat{h}$(***z***). We hence summarized the goodness-of-fit of $\hat{h}$(·) in the following way. For each simulated data set, we regressed the true *h *on the fitted value $\hat{h}$, both evaluated at the design points. We then empirically summarized the goodness-of-fit of $\hat{h}$(·) by calculating the average intercepts, slopes and *R*^2^'s obtained from these regressions over the 300 simulations. If the kernel machine method fits the nonparametric function well, then we would expect the intercept to be close to 0, the slope close to 1, and *R*^2^also close to 1.

**Table 2:** Simulation results on estimation.

				Model Parameter Estimates		Reg of *h *on $\hat{h}$		
					
setting	true # *z*	used # *z*	*n*	*β*	*ρ*	Intercept	Slope	*R*^2^
1	5	5	100	1.10	71.50^*a*^(estimated)	-0.06	1.06	0.82
				1.14	1.00 (fixed)	-0.28	1.48	0.79
				1.08	20.00 (fixed)	-0.08	1.15	0.84
2	5	5	200	0.99	90.03 (estimated)	0.01	1.04	0.87
				1.05	1.00 (fixed)	-0.01	1.13	0.84
				0.96	20.00 (fixed)	-0.00	1.07	0.87
3	5	5	300	0.98	111.76 (estimated)	-0.01	1.04	0.90
				1.03	1.00 (fixed)	-0.02	1.10	0.87
				0.97	20.00 (fixed)	-0.01	1.06	0.90

This table shows the simulation results of estimated regression coefficients *β*and the nonparametric function *h*(·) in model logit(*π*) = *xβ*+ *h*(*z*) for binary outcomes based on 300 runs. True *β *= 1. In the table, ^*a *^is the average of the estimated $\hat{\rho }$ from 300 simulations.

Our results show that even when the sample size is as low as 100, estimation of the regression coefficient and nonparametric function only has small bias. When the kernel parameter *ρ *is estimated, these biases tend to be small compared with those when *ρ *is held fixed. With the increase of sample size the estimates of *β *and *h *become closer to the true values, especially when *ρ *is estimated, while there are still some bias when *ρ *is fixed at values farther away from the estimated one. Table 3 compares the estimated standard errors of $\hat{\beta }$ with the empirical standard errors. Our results show that they agree to each other well when *ρ *is estimated.

**Table 3:** Simulation results on standard errors.

				Standard Errors of $\hat{\beta }$		
					
	true	used		Empirical	Model-based	
setting	# *z*	# *z*	*n*	SE	SE	*ρ*
1	5	5	100	0.49	0.48	71.50 (estimated)
				0.45	0.47	1.00 (fixed)
				0.48	0.47	20.00 (fixed)
2	5	5	200	0.32	0.32	90.03 (estimated)
				0.32	0.32	1.00 (fixed)
				0.33	0.32	20.00 (fixed)
3	5	5	300	0.26	0.26	111.76 (estimated)
				0.25	0.26	1.00 (fixed)
				0.26	0.26	20.00 (fixed)

This table shows the simulation study results of standard error estimates of $\hat{\beta }$ in model logit(*π*) = *xβ*+ *h*(*z*) for binary outcomes based on 300 simulations.

### Simulation Study of the Score Test for the Pathway Effect

We next conducted a simulation study to evaluate the performance of the proposed variance component score test for the pathway effect *H*_0_: *h*(·) = 0 vs *H*_1_: *h*(·) ≠ 0. In order to compare the performance of our test with the linearity-based global test proposed by Goeman et al. [13], both tests were applied to each simulated data set. Nonlinear and linear functions of *h*(***z***) were both considered. For the nonlinear pathway effect, the true model is logit(*y*) = *x *+*ah*(***z***), where *h*(***z***) = 2(*z*_1 _- *z*_2_)^2 ^+ *z*_2_*z*_3 _+ 3 sin(2*z*_3_)*z*_4 _+ $z_{5}^{2}$ + 2 cos(*z*_4_)*z*_5_. For the linear pathway effect, the true model is logit(*y*) = *x *+ *ah*(***z***), where *h*(***z***) = 2*z*_1 _+ 3*z*_2_+*z*_3 _+ 2*z*_4_+*z*_5_. All *z*'s were generated from the standard normal distribution, and *a *= 0, 0.2, 0.4, 0.6, 0.8. To allow *x *and (*z*_*i*1_, ⋯, *z*_*ip*_) to be correlated, *x *was generated as *x *= *z*_1_+*e*/2 with *e *being independent of *z*_1 _and following *N *(0, 1). We studied the size of the test by generating data under *a *= 0, and studied the power by increasing *a*. The sample size was 100. For the size calculations, the number of simulations was 2000; whereas for the power calculations, the number of runs was 1000. Based on the discussions in Section "Test for the genetic Pathway Effect", the bound of *ρ *is set up by interval $\left[\min_{i\neq j}\sum_{l=1}^{5}{\left(z_{il}-z_{jl}\right)}^{2}/5,10\max_{i\neq j}\sum_{l=1}^{5}{\left(z_{il}-z_{jl}\right)}^{2}\right]$, and the interval is divided by 500 equally spaced grid points. All simulations were conducted using R 2.5.0, and the package "globaltest" v4.6.0 was used for the test proposed by Goeman et al. [13] as a comparison.

Table 4 reports the empirical size (*a *= 0) and power (*a *> 0) of the variance component score test for the pathway effect. When the true function *h*(***z***) is non-linear in ***z***, the results show that the size of our test was very close to the nominal value 0.05, while the size of the global test of Goeman et al. [13] is inflated. The results also show that our test had a much higher power. This was not surprising since the test of Goeman et al. [13] was based on a linearity assumption of the pathway effect. When the true underlying model is far from linear, the linearity assumption breaks down and the test quickly loses power. The results also show that the proposed test works well for moderate sample sizes. When the pathway effect is linear, the results show that the size of both tests were very close to the nominal value 0.05 and their power were also very close. This demonstrates that our test is as powerful as the global test when the true underlying *h*(***z***) is linear. Therefore our test could be used as a universal test for testing the overall effect of a set of variables without the need to specify the true functional forms of each variable. This feature is especially desirable for genetic pathway data, because the relationship between genes and clinical outcome is often unknown.

**Table 4:** Simulation results on score test.

		Size	Power		
			
*h*(***z***)	Method	*a *= 0	*a *= 0.2	*a *= 0.4	*a *= 0.8
Nonlinear	KM	0.054	0.142	0.896	1.000
	GT	0.068	0.098	0.110	0.156
Linear	KM	0.055	0.265	0.896	1.000
	GT	0.065	0.302	0.900	1.000

This table shows the simulation study results of standard error estimates of $\hat{\beta }$ in model logit(*π*) = *xβ*+ *h*(*z*) for binary outcomes based on 300 simulations.

## Conclusions and Discussion

In this paper, we developed a logistic kernel machine regression model for binary outcomes, where the covariate effects are modeled parametrically and the genetic pathway effect is modeled nonparametrically using the kernel machine method. This method provides an attractive way to model the pathway effect, without the need to make strong parametric assumptions on individual gene effects or their interactions. Our model also allows for parametric pathway effects if a parametric kernel, such as the first-degree polynomial kernel, is used.

A key result of this paper is that we have established a close connection between the generalized kernel machine regression and generalized linear mixed models, and show that the kernel machine estimators of regression coefficients and the nonparametric multi-dimensional pathway effect can be easily obtained from the corresponding generalized linear mixed models using PQL. The mixed model connection provides a unified framework for estimation and inference and can be easily implemented in existing software, such as SAS PROC GLIMMIX or R GLMMPQL. The mixed model connection also makes it possible to test for the overall pathway effect through the proposed variance component test. A key advantage of the proposed score test for the pathway effect is that it does not require an explicit functional specification of individual gene effects and gene-gene interactions. This feature is of practical significance as the pathway effect is often complex. Our simulation study shows the proposed test performs well for moderate sample size. It has similar power to the linearity-based pathway test of Goeman et al. [13] when the true effect is linear, but much higher power when the true effect is nonlinear.

We have considered in this paper a single pathway. One could generalize the proposed semiparametric model to incorporate multiple pathways by fitting an additive model:

logit(*P*(*y *= 1)) = ***x***^*T*^***β ***+ *h*_1_(***z***_1_) + ⋯ + *h*_*m*_(***z***_*m*_),

where ***z***_*j *_(*j *= 1, ⋯, *m*) denotes a *p*_*j *_× 1 vector of genes in the *j*th pathway and *hj*(·) denotes the nonparametric function associated with the *j*th genetic pathway.

Machine learning is a powerful tool in advancing bioinformatics research. Our effort helps to build a bridge between kernel machine methods and traditional statistical models. This connection will undoubtedly provide a new and convenient tool for the bioinformatics community and opens a door for future research.

## Methods

### The Logistic Kernel Machine Model

Throughout the paper we assume that gene expression data have been properly normalized. Suppose the data consist of *n *samples. For subject *i *(*i *= 1, ⋯, *n*), *y*_*i *_is a binary disease outcome taking values either 0 (non-disease) or 1 (disease), ***x***_*i *_is a *q *× 1 vector of covariates, ***z***_*i *_is a *p *× 1 vector of gene expression measurements in a pathway/gene set. We assume that an intercept is included in ***x***_*i*_. The binary outcome *y*_*i *_depends on ***x***_*i *_and ***z***_*i *_through the following semiparametric logistic regression model:

(3) $$\text{logit}(\mu_{i})=x_{i}^{T}\beta +h(z_{i}),$$

where *μ*_*i *_= *P *(*y*_*i *_= 1| ***x***_*i*_, ***z***_*i*_), ***β ***is a *q *× 1 vector of regression coefficients, and *h*(***z***_*i*_) is an unknown centered smooth function.

In model (3), covariate effects are modeled parametrically, while the multi-dimensional genetic pathway effect is modeled parametrically or nonparametrically. A nonparametric specification for *h*(·) reflects our limited knowledge of genetic functional forms. Note that *h*(·) = 0 means that genes in the pathway have no association with the disease risk. If *h*(***z***) = *γ*_1_*z*_1 _+ ⋯ + *γ*_*p*_*z*_*p*_, model (3) becomes the linear model considered by Goeman et al. [13].

In nonparametric modeling, such as smoothing splines, the unknown function is usually assumed to lie in a certain function space. For the kernel machine method, this function space, denoted by $H_{K}$, is generated by a given positive definite kernel function *K*(·, ·). The mathematical properties of $H_{K}$ imply that any unknown function *h*(***z***) in $H_{K}$ can be written as a linear combination of the given kernel function *K*(·, ·) evaluated at each sample point. Two popular kernel functions are the *d*th polynomial kernel $K(z_{1},z_{2})={\left(z_{1}^{T}z_{2}+\rho \right)}^{d}$ and the Gaussian Kernel *K*(***z***_1_, ***z***_2_) = exp{-|| ***z***_1 _– ***z***_2_||^2^/*ρ*^2^}, where $||z_{1}-z_{2}|{|}^{2}=\sum_{k=1}^{p}{\left(z_{1k}-z_{2k}\right)}^{2}$ and *ρ *is an unknown parameter. The first and second degree polynomial kernels (*d *= 1, 2) correspond to assuming *h*(·) to be linear and quadratic in *z*'s, respectively. The choice of a kernel function determines which function space one would like to use to approximate *h*(***z***). The unknown parameter of a kernel function plays a critical role in function approximation. It is a challenging problem to optimally estimate it from data. In the machine learning literature, this parameter is usually pre-fixed at some values based on some ad-hoc methods. In this paper, we show that we can optimally estimate it from data based on a mixed model framework.

### The Estimation Procedure

Assuming *h*(·) ∈ $H_{K}$, the function space generated by a kernel function *K*(·, ·), we can estimate *β *and *h*(·) by maximizing the penalized log-likelihood function

(4) $$\begin{array}{lll} J(h,\beta ) & = & \sum_{i=1}^{n}\left\{y_{i}\log\left(\frac{\mu_{i}}{1-\mu_{i}}\right)+\log(1-\mu_{i})\right\}-\frac{1}{2}\lambda ||h|{|}_{H_{K}}^{2}\\ & = & \sum_{i=1}^{n}(y_{i}\{x_{i}^{T}\beta +h(z_{i})\}-\log[1+\exp\{x_{i}^{T}\beta +h(z_{i})\}])-\frac{\lambda }{2}||h|{|}_{H_{K}}^{2}, \end{array}$$

where *λ *is a regularization parameter that controls the tradeoff between goodness of fit and complexity of the model. When *λ *= 0, it fits a saturated model, and when *λ *= ∞, the model reduces to a simple logistic model logit (*μ*_*i*_) = $x_{i}^{T}\beta$. Note that there are two tuning parameters in the above likelihood function, the regularization parameter *λ *and kernel parameter *ρ *Intuitively, *λ *controls the magnitude of the unknown function while *ρ *mainly governs the smoothness property of the function.

By the representer theorem [16], the general solution for the nonparametric function *h*(·) in (4) can be expressed as

(5) $$h(z_{i})=\sum_{i^{\prime }=1}^{n}\alpha_{i^{\prime }}K(z_{i},z_{i^{\prime }})=k_{i}^{T}\alpha ,$$

where ***k***_*i *_= {*K*(***z***_*i*_, ***z***_1_), ..., *K*(***z***_*i*_, ***z***_*n*_)}^*T *^and ***α ***= (*α*_1_, ⋯, *α*_*n*_)^*T*^, an *n *× 1 vector of unknown parameters.

Substituting (5) into (4) we have

(6) $$J(\beta ,\alpha )=\sum_{i=1}^{n}\left[y_{i}(x_{i}^{T}\beta +k_{i}^{T}\alpha )-\log\left\{1+\exp\left(x_{i}^{T}\beta +k_{i}^{T}\alpha \right)\right\}\right]-\frac{1}{2}\lambda \alpha^{T}K\alpha ,$$

where ***K ***= ***K***(*ρ*) is an *n *× *n *matrix whose (*i*, *i'*)th element is *K*(***z***_*i*_, ***z***_*i*_*'*) and often depends on an unknown parameter *ρ*.

Since *J*(***β***, ***α***) in (6) is a nonlinear function of (***β***, ***α***), one can use the Fisher scoring or Newton-Raphson iterative algorithm to maximize (6) with respect to ***β ***and ***α***. Let (*k*) denote the *k*^th ^iteration step, then it can be shown (for details see Appendix A.3) that the (*k *+ 1)^th ^update for ***β ***and ***α ***solves the following normal equation:

(7) $$\left[\begin{array}{cc} X^{T}D^{\left(k\right)}X & X^{T}D^{\left(k\right)}K\\ D^{\left(k\right)}X & \tau^{-1}I+D^{\left(k\right)}K \end{array}\right]\;\left[\begin{array}{c} \beta^{\left(k+1\right)}\\ \alpha^{\left(k+1\right)} \end{array}\right]=\left[\begin{array}{c} X^{T}D^{\left(k\right)}{\tilde{y}}^{\left(k\right)}\\ D^{\left(k\right)}{\tilde{y}}^{\left(k\right)} \end{array}\right].$$

where ${\tilde{y}}^{\left(k\right)}=X\beta^{\left(k\right)}+K\alpha^{\left(k\right)}+D^{{\left(k\right)}^{-1}}(y-\mu^{\left(k\right)})$, *τ *= 1/*λ*, ***h***^(*k*) ^= ***Kα ***^(*k*)^, and $D^{\left(k\right)}=\text{Diag}\{\mu_{i}^{\left(k\right)}(1-\mu_{i}^{\left(k\right)})\}$. The estimators $\hat{\beta }$ and $\hat{h}$ at convergence are the kernel machine estimators that maximize (6).

### The Connection of Logistic Kernel Machine Regression to Logistic Mixed Models

Generalized linear mixed models (GLMMs) have been used to analyze correlated categorical data and have gained much popularity in the statistical literature [10]. Logistic mixed models are a special case of GLMMs. We show in this section that the kernel machine estimator in the semiparametric logistic regression model (3) corresponds to the Penalized Quasi-Likelihood (PQL) [10] estimator from a logistic mixed model, and the regularization parameter *τ *= 1/*λ *and kernel parameter *ρ *can be treated as variance components and estimated simultaneously from the corresponding logistic mixed model. Specifically, consider the following logistic mixed model:

(8) $$\text{logit}(\mu_{i})=x_{i}^{T}\beta +h_{i},$$

where ***β ***is a *q *× 1 vector of fixed effects, and ***h ***= (*h*_1_, ..., *h*_*n*_) is a *n *× 1 vector of subject-specific random effects following ***h ****~N*{**0**, *τ ****K***(*ρ*)}, and the covariance matrix ***K***(*ρ*) is the *n *× *n *kernel matrix as defined in previous section.

As ***K ***is not diagonal or block-diagonal, the random effects *h*_*i*_'s across all subjects are correlated. The *i*^th ^mean response *μ*_*i *_depends on other random effects *h*_*i*_*' *(*i*' ≠ *i*) through the correlations of *h*_*i *_with other random effects. To estimate the unknown parameters in the logistic mixed model (8), we estimate ***β ***and ***h ***by maximizing the PQL [10], which can be viewed as a joint log likelihood of (***β ***, ***h***),

(9) $$\sum_{i=1}^{n}\left[y_{i}(x_{i}^{T}\beta +h_{i})-\log\{1+\exp(x_{i}^{T}\beta +h_{i})\}\right]-\frac{1}{2\tau }h^{T}K^{-1}h.$$

Setting *τ *= 1/*λ *and ***h ***= ***Kα ***, one can easily see that equations (6) and (9) are identical. It follows that the logistic kernel machine estimators $\hat{\beta }$ and $\hat{h}$ can be obtained by fitting the logistic mixed model (8) using PQL. In fact, examination of the kernel machine normal equations (7) shows that they are identical to the normal equations obtained from the PQL (9) [10], where $\tilde{y}$ in (7) is in fact the PQL working vector and ***D***is the PQL working weight matrix.

Note that the estimators of ***β ***and ***h ***depend on the unknown regularization parameter *τ *and the kernel parameter *ρ*. Within the PQL framework, we can estimate these parameters ***δ ***= (*τ*, *ρ*) by maximizing the approximate REML likelihood

(10) $$\ell_{R}(\hat{\beta }(\delta ),\delta )\approx -\frac{1}{2}\log|V|-\frac{1}{2}\log|X^{T}V^{-1}X|-\frac{1}{2}{\left(\tilde{y}-X\hat{\beta }\right)}^{T}V^{-1}(\tilde{y}-X\hat{\beta }),$$

where ***V ***= ***D***^-1 ^+ *τ ****K***, and $\tilde{y}$ is the working vector as defined above. The estimator $\hat{\delta }$ of ***δ ***can be obtained by setting equal to zero the first derivative of (10) with respect to ***δ***. The estimating procedure for ***β***, ***h***, and ***δ ***= (*τ*, *ρ*) can be summarized as follows: we fit the logistic kernel machine model by iteratively fitting the following working linear mixed model to estimate (***β***, ***h***) using BLUPs and to estimate ***δ ***using REML, until convergence

$$\tilde{y}=X\beta +h+\varepsilon ,$$

where $\tilde{y}$ is the working vector defined below equation (7), ***h ***is a random effect vector following *N*{0, *τ ****K***(*ρ*)}, and **ε **~ *N*(0, ***D***). The covariance of $\hat{\beta }$ is estimated by (***X***^*T*^***V***^-1^***X***)^-1^, and the covariance of $\hat{h}$ is estimated by $\hat{\tau }K-\hat{\tau }KPK$, where ***P ***= ***V***^-1 ^- ***V***^-1^***X***(***X***^*T*^***V***^-1^***X***)^-1^***X***^*T*^***V***^-1 ^and ***V ***= ***V***($\hat{\delta }$). The covariance of $\hat{\delta }$ can be obtained as the inverse of the expected information matrix calculated using the second derivative of (10) with respect to ***δ***. The square roots of the diagonal elements of the estimated covariance matrices give the standard errors of $\hat{\beta }$, $\hat{h}$, and ***δ***. The above discussion shows that we can easily fit the logistic kernel machine regression using the existing PQL-based mixed model software, such as SAS GLIMMIX and R GLMMPQL.

### Test for the Genetic Pathway Effect

It is of significant practical interest to test the overall genetic pathway effect *H*_0_: *h*(***z***) = 0. Assuming *h*(***z***) ∈ $H_{K}$, one can easily see from the logistic mixed model representation (8) that *H*_0_: *h*(***z***) = 0 vs *H*_1_: *h*(***z***) ≠ 0 is equivalent to testing the variance component *τ *as *H*_0_: *τ *= 0 vs *H*_1_: *τ *> 0. Note that the null hypothesis places *τ *on the boundary of the parameter space. Since the kernel matrix ***K ***is not block diagonal, unlike the standard case considered by Self and Liang [17], the likelihood ratio for *H*_0_: *τ *= 0 does not follow a mixture of $\chi_{0}^{2}$ and $\chi_{1}^{2}$ distribution. We consider instead a score test in this paper.

When conducting statistical tests for pathways, two types of tests could be formulated. The first is called the competitive test and the second the self-contained test [18]. The competitive test compares an interested gene set to all the other genes on a gene chip. An example of the competitive test is the gene set enrichment analysis (GSEA) [1], where an enrichment score of a gene set is defined and a permutation test is used to test for the significance of the gene set based on the enrichment score. The self-contained test compares the gene set to an internal standard which does not involve any genes outside the gene set considered. In other words, the self-contained test examines the null hypothesis that a pathway has no effect on the outcome versus the alternative hypothesis that the pathway has an effect. The variance component test of [13] for the linear pathway effect is a self-contained test. Goeman and Bühlmann [18] pointed out that the self-contained test has a higher power than a competitive test and that its statistical formulation is also consistent for both single gene tests and gene set tests, and the statistical sampling properties of the competitive test can be difficult to interpret.

Our pathway effect hypothesis *H*_0_: *h*(***z***) = 0 vs *H*_1_: *h*(***z***) ≠ 0 is a self-contained hypothesis. We propose in this paper a self-contained test for the pathway effect by developing a kernel machine variance component score test for *H*_0_: *τ *= 0 vs *H*_0_:*τ *> 0. The proposed test allows for both linear and nonlinear pathway effects and includes the tests by Goeman et al. [13,14] as a special case. A key advantage of our kernel-based test is that we do not need to explicitly specify the basis functions for *h*(·), which is often difficult for modeling the joint effects of multiple genes, and we all let the data to estimate the best curvature of *h*(·).

Zhang and Lin [19] proposed a score test for *H*_0_: *τ *= 0 to compare a polynomial model with a smoothing spline. Goeman et al. [14] also proposed a global test against a high dimensional alternative under the empirical Bayesian framework. The variance-covariance matrix used in these tests do not involve any unknown parameters. However, the kernel function *K*(·, ·) in a kernel machine model usually depends on some unknown parameter *ρ*. One can easily see from the mixed model representation (8) that under *H*_0_: *τ *= 0, the kernel matrix ***K ***disappears. This makes the parameter *ρ *inestimable under the null hypothesis and therefore renders the above tests inapplicable.

Davies [20,21] studied the problem of a parameter disappearing under *H*_0 _and proposed a score test by treating the score statistic as a Gaussian process indexed by the nuisance parameter and then obtaining an upper bound to approximate the p-value of the score test. We adopt this line of approaches for our proposed score test.

Using the derivative of (10) with respect to *τ*, we propose the following score test statistic for *H*_0_: *τ *= 0 as

(11) $$S(\rho )=\frac{Q_{\tau }(\overset{\wedge }{\beta_{0}},\rho )-\mu_{Q}}{\sigma_{Q}},$$

where

$$Q_{\tau }({\hat{\beta }}_{0},\rho )={\left(\tilde{y}-X{\hat{\beta }}_{0}\right)}^{T}DK(\rho )D(\tilde{y}-X{\hat{\beta }}_{0})={\left(y-{\hat{\mu }}_{0}\right)}^{T}K(y-{\hat{\mu }}_{0}),$$

where ${\hat{\beta }}_{0}$ is the MLE of ***β ***under *H*_0_: *τ *= 0, ${\hat{\mu }}_{0}=logit^{-1}(X{\hat{\beta }}_{0})$, *μ*_*Q *_= tr{***P***_0_***K***(*ρ*)}, $\sigma_{Q}^{2}$ = 2tr{***P***_0_***K***(*ρ*)***P***_0_***K***(*ρ*)}, and ***P***_0 _= ***D***_0 _– ***D***_0_***X***(***X***^*T*^***D***_0_***X***)^-1 ^***X***^*T*^***D***_0_, where $D_{0}=\text{diag}\{{\hat{\mu }}_{i0}(1-\mu_{i0})\}$. Note that under *H*_0_: *τ *= 0, model (3) reduces to the simple logistic model *logit *(*μ*_*i*_) = $x_{i}^{T}\beta$. Hence the ${\hat{\beta }}_{0}$ is the MLE of ***β ***under this null logistic model.

If the Gaussian kernel is used, then an arbitrary nonlinear pathway effect is implicitly assumed. Our proposed test, which is derived to test for any nonlinear effect, is therefore more powerful than tests based on a parametric assumption. We show in Appendix A.1 that when *ρ *becomes large in the Gaussian kernel, our test statistic reduces asymptotically to the one based on linearity assumption of genetic effects. Hence our test includes linear model based test as a special case. From (11) it is also clear that our test is invariant to the relative scaling of the kernel function *K*(·, ·).

Under appropriate regularity conditions similar to those specified in [22], *S*(*ρ*) under the null hypothesis can be considered as an approximate Gaussian process indexed by *ρ*. Using this formulation, we can then apply Davies' results [20,21] to obtain the upper bound for the p-value of the test. Since a large value of *Q*_τ _($\hat{\beta }$, *ρ*) would lead to the rejection of *H*_0_, the p-value of the test corresponds to the up-crossing probability. Following Davies [21], the p-value is upper-bounded by

(12) $$\Phi (-M)+W\exp(-\frac{1}{2}M^{2})/\sqrt{8\pi },$$

where Φ (·) is the normal cumulative distribution function, *M *is the maximum of *S*(*ρ*) over the range of *ρ*, *W *= | *S*(*ρ*_1_) – *S*(*L*)| + | *S*(*ρ*_2_) – *S*(*ρ*_1_) | + ... + | *S*(*U*) – *S*(*ρ*_*m*_) |, *L *and *U *are the lower and upper bound of *ρ *respectively and *ρ*_*l*_, *l *= 1, ..., *m *are the *m *grid points between *L *and *U*. Davies [20] points out that this bound is sharp. For the Gaussian kernel, we suggest to set the bound of *ρ *as *L *= 0.1 $\min_{i\neq j}\sum_{l=1}^{p}{\left(z_{il}-z_{jl}\right)}^{2}$ and *U *= 100 $\max_{i\neq j}\sum_{l=1}^{p}{\left(z_{il}-z_{jl}\right)}^{2}$. For justifications, see Appendix A.2.

### Extension to generalized kernel machine model

For simplicity, we focus in this paper on logistic regression for binary outcomes. The proposed semiparametric model (3) can be easily extended to other types of continuous and discrete outcomes, such as normal, count, skewed data, whose distributions are in the exponential family [23]. In this section, we briefly discuss how to generalize our estimation and testing procedures for binary outcomes to other data types within the generalized kernel machine framework and discuss its fitting using generalized linear mixed models.

Suppose the data consist of *n *independent subjects. For subject *i *(*i *= 1, ..., *n*), *y*_*i *_is a response variable, ***x***_*i *_is a *q *× 1 vector of covariates, ***z***_*i *_is a *p *× 1 vector of gene expressions within a pathway. Suppose *y*_*i *_follows a distribution in the exponential family with density [23]

(13) $$p(y_{i};\theta_{i},\varphi )=\exp\left\{\frac{y_{i}\theta_{i}-a(\theta_{i})}{\varphi /m_{i}}+c(y_{i},\varphi )\right\},$$

where *θ*_*i *_is the canonical parameter, *a*(·) and *c*(·) are known functions, *φ *is a dispersion parameter, and *m*_*i *_is a known weight. The mean of *y*_*i *_satisfies *μ*_*i *_= *E*(*y*_*i*_) = *a*'(*θ*_*i*_) and *Var*(*y*_*i*_) = *φ m*_*i*_*a"*(*θ*_*i*_). The generalized kernel machine model is an extension of the generalized linear model [23] by allowing the pathway effect to be modeled nonparametrically using kernel machine as

(14) $$g(\mu_{i})=x_{i}^{T}\beta +h(z_{i}),$$

where *g*(·) is a known monotone link function, and *h*(·) is an unknown centered smooth function lying in the function space $H_{K}$ generated by a positive definite kernel function *K*(·, ·). For binary data, setting *g*(*μ*) = logit(*μ*) = $\log\frac{\mu }{1-\mu }$ gives the logistic kernel machine model (3); for count data, *g*(*μ*) = log(*μ*) gives the Poisson kernel machine model; for Gaussian data, *g*(*μ*) = *μ *gives linear kernel machine model [9]. The regression coefficients ***β ***and the nonparametric function *h*(·) in (14) can be obtained by maximizing the penalized log-likelihood function

(15) $$J(h,\beta )=\sum_{i=1}^{n}\ell \{y_{i},x_{i},z_{i};\beta ,h(\cdot )\}-\frac{1}{2}\lambda ||h|{|}_{H_{K}}^{2}$$

where ℓ(·) = *ln*(*p*) is the log-likelihood, *p *is the density function given in (13), and *λ *is a tuning parameter. Using the kernel expression of *h*(·) in (5), the generalized kernel machine model (14) can be written as

$$g(\mu_{i})=x_{i}^{T}\beta +k_{i}^{T}\alpha ,$$

and the penalized likelihood can be written

(16) $$J(\beta ,\alpha )=\sum_{i=1}^{n}\ell (y_{i},x_{i},z_{i};\beta ,\alpha )-\frac{1}{2}\lambda \alpha^{T}K\alpha ,$$

where ***K ***is an *n *× *n *matrix whose (*i*, *j*)th element is *K*(***z***_*i*_, ***z***_*j*_).

One can use the Fisher scoring iteration to solve for ***β ***and ***a***. The procedure is virtually the same as that described in Section "The Estimation Procedure". The normal equation takes the same form as (7), except that now *μ*_*i *_is specified under (14) and ***D ***= *diag*{*var*(*y*_*i*_)} under (13). Similar calculations to those in Section "The Connection of Logistic Kernel Machine Regression to Logistic Mixed Models" show that model (14) can be fit using the generalized linear mixed model [10] via PQL

$$g(\mu_{i}^{b})=x_{i}^{T}\beta +h_{i},$$

where *τ *= 1/*λ*, and ***h ***= (*h*_1 _..., *h*_*n*_) is an *n × n *random vector with distribution *N *{0, *τ ****K***(*ρ*)}. The same PQL statistical software, such as SAS PROC GLIMMIX and R GLMMPQL, can be used to fit this model and obtain the kernel machine estimators of ***β ***and *h*(·).

The score test (11) also has a straightforward extension. The only change is that the elements in matrix ***D ***in (11) be replaced by appropriate variance function *var*(*y*_*i*_) under the assumed parametric distribution of *y*_*i*_.

## Appendix

### A.1 Proof of the relationship of the proposed score test and that of Goeman, et al [13] under the linearity assumption

We show in this section when the scale parameter is large, the proposed nonparametric variance component test for the pathway effect using the Gaussian kernel reduces to the linearity-based global test of Goeman et al. [13].

Suppose *K*(·) is the Gaussian kernel. It can be shown that the score statistic for testing *H*_0_: *τ *= 0 satisfies

(17) $$Q_{\tau }(\hat{\beta },\rho )={\left(\tilde{y}-X{\hat{\beta }}_{0}\right)}^{T}DK(\rho )D(\tilde{y}-X{\hat{\beta }}_{0})={\left(y-\hat{\mu }\right)}^{T}K(\rho )(y-{\hat{\mu }}_{0}),$$

where ${\hat{\mu }}_{0}$ is the MLE of ***μ ***under *H*_0_. The test statistic of Goeman et al. [13] takes the form

(18) $${\left(y-\hat{\mu }\right)}^{T}R(h-\hat{\mu }),$$

where ***R ***= ***ZZ***^*T*^. We now show when *ρ *is large relative to $\max_{i\neq j}\sum_{l=1}^{p}{\left(z_{il}-z_{jl}\right)}^{2}$

(19) $$\frac{\rho }{2}{\left(y-\hat{\mu }\right)}^{T}K(\rho )(y-\hat{\mu })\approx {\left(y-\hat{\mu }\right)}^{T}R(y-\hat{\mu }).$$

Simple Taylor expansions show that

$$\begin{array}{lll} {\left(y-\hat{\mu }\right)}^{T}K(\rho )(y-\hat{\mu }) & = & \sum_{i=1}^{n}\sum_{j=1}^{n}(y_{i}-{\hat{\mu }}_{i})(y_{j}-{\hat{\mu }}_{j})\exp\{-\sum_{l=1}^{p}{\left(z_{il}-z_{jl}\right)}^{2}/\rho \}\\ & = & \sum_{i=1}^{n}{\left(y_{i}-{\hat{\mu }}_{i}\right)}^{2}+\sum_{i\neq j}(y_{i}-{\hat{\mu }}_{i})(y_{j}-{\hat{\mu }}_{j})\exp\{-\sum_{l=1}^{p}{\left(z_{il}-z_{jl}\right)}^{2}/\rho \}. \end{array}$$

When $\max_{i\neq j}\sum_{l=1}^{p}{\left(z_{il}-z_{jl}\right)}^{2}/\rho$ is small, i.e., when *ρ *is large relative to $\max_{i\neq j}\sum_{l=1}^{p}{\left(z_{il}-z_{jl}\right)}^{2}$, we have that $\exp\{-\sum_{l=1}^{p}{\left(z_{il}-z_{jl}\right)}^{2}/\rho \}\approx 1-\sum_{l=1}^{p}{\left(z_{il}-z_{jl}\right)}^{2}/\rho$ for any *i *≠ *j*. Hence

$$\begin{array}{ll} & {\left(y-\hat{\mu }\right)}^{T}K(\rho )(y-\hat{\mu })\\ = & \sum_{i=1}^{n}{\left(y_{i}-{\hat{\mu }}_{i}\right)}^{2}+\sum_{i\neq j}\left(y_{i}-{\hat{\mu }}_{i})(y_{j}-{\hat{\mu }}_{j}\right)-\frac{1}{\rho }\sum_{i\neq j}\left(y_{i}-{\hat{\mu }}_{i})(y_{j}-{\hat{\mu }}_{j}\right)\sum_{l=1}^{p}{\left(z_{il}-z_{jl}\right)}^{2}. \end{array}$$

Since $\sum_{j=1}(y_{j}-{\hat{\mu }}_{j})=0$ under the PQL, we have $\sum_{j\neq 1}\left(y_{j}-{\hat{\mu }}_{j})=-(y_{i}-{\hat{\mu }}_{i}\right)$. Hence

$$\begin{array}{ll} & {\left(y-\hat{\mu }\right)}^{T}K(\rho )(y-\hat{\mu })\\ \approx & \sum_{i=1}^{n}{\left(y_{i}-{\hat{\mu }}_{i}\right)}^{2}-\sum_{i=j}^{n}{\left(y_{i}-{\hat{\mu }}_{i}\right)}^{2}-\frac{1}{\rho }\sum_{i\neq j}\left(y_{i}-{\hat{\mu }}_{i})(y_{j}-{\hat{\mu }}_{j}\right)\sum_{l=1}^{p}\left(z_{il}^{2}-2z_{il}z_{jl}+z_{jl}^{2}\right)\\ = & \frac{2}{\rho }\sum_{i=1}^{n}{\left(y_{i}-{\hat{\mu }}_{i}\right)}^{2}\sum_{l=1}^{p}z_{il}^{2}+\frac{2}{\rho }\sum_{i\neq j}\left(y_{i}-{\hat{\mu }}_{i})(y_{j}-{\hat{\mu }}_{j}\right)\sum_{l=1}^{p}z_{il}z_{jl}\\ = & \frac{2}{\rho }{\left(y-\hat{\mu }\right)}^{T}R(y-\hat{\mu }). \end{array}$$

This proves the approximate relation (19).

### A.2 Calculations of the lower and upper bounds of *ρ*

Although in theory *ρ *could take any positive values up to infinity, for computational purpose we would require *ρ *to be bounded. For the proposed test statistic (11), its value in fact only depends on a finite range of *ρ *values. We describe why this is the case and how to find this range. For a given data set, the proof in Appendix A.1 shows that when is sufficiently large, the quantity 0.5*ρQ*_*τ *_(${\hat{\beta }}_{0}$, *ρ*) converges to $S_{0}={\left(\tilde{y}-{\hat{\mu }}_{0}\right)}^{T}R(\tilde{y}-{\hat{\mu }}_{0})$, which is free of *ρ*.

These arguments suggest that for numerical evaluation, it is not necessary to consider all *ρ *values up to infinity. Instead, a moderately large enough value would suffice. Now the question comes down to how to decide on appropriate upper and lower bounds for *ρ*. The proof in Appendix A.1 requires $\max_{i\neq j}\sum_{l=1}^{p}{\left(z_{il}-z_{jl}\right)}^{2}/\rho$ be close to 0. Let *C*_1 _be some large positive number such that 1/*C*_1 _≈ 0. If we take the upper bound of *ρ *to be *C*_1 _$\max_{i\neq j}\sum_{l=1}^{p}{\left(z_{il}-z_{jl}\right)}^{2}$, then $\max_{i\neq j}\sum_{l=1}^{p}{\left(z_{il}-z_{jl}\right)}^{2}/\rho$ would be close to 0. In practice we suggest taking *C*_1 _= 100, which would give good approximation. Using a similar idea, we can find a lower bound for *ρ*. It is clear that when $\min_{i\neq j}\sum_{l=1}^{p}{\left(z_{il}-z_{jl}\right)}^{2}$/*ρ *→ ∲ any non-diagonal element of ***K***(*ρ*) will be 0 and the kernel matrix reduces to an identity matrix. Hence, if we pick a small enough number *C*_2 _such that 1/*C*_2 _→ ∲, we can effectively set the lower bound of *ρ *to be *C*_2 _$\min_{i\neq j}\sum_{l=1}^{p}{\left(z_{il}-z_{jl}\right)}^{2}$. In practice we suggest take *C*_2 _= 0.1, which yields a good approximation.

### A.3 derivation of normal equation (5)

Taking partial derivative of (6) with respect to ***β ***and writing in matrix notation, we have ***X***^*T*^(***y***-***μ***). Similarly for ***α***, we have ***K***(***y ***– ***μ***) – *λ ****Kα***. The gradient vector is thus

(20) $$q=\left[\begin{array}{c} X^{T}(y-\mu )\\ K(y-\mu )-\lambda K\alpha \end{array}\right].$$

Taking derivative of ***q ***with respect to ***β ***and ***α***, we can get the following hessian matrix

(21) $$H=-\left[\begin{array}{cc} X^{T}DX & X^{T}DK\\ KDX & \lambda K+KDK \end{array}\right],$$

where ***D ***= Diag{*μ*_*i*_(1 – *μ*_*i*_)}. The Newton-Raphson iteration states that the parameter value at the (*k *+ 1)^th ^iteration can be updated by the following relationship

(22) ***δ***^(*k*+1) ^= ***δ***^(*k*) ^– (*H*^(*k*)^)^-1 ^***q***^(*k*)^,

where ***δ ***= (***β***^*T*^, ***α***^*T*^)^*T*^. Substitute (20) and (21) into (22), we arrive at normal equation (7).

## Availability

Our algorithm is available at .

## Authors' contributions

DL performed statistical analysis. All authors participated in development of the methods and preparation of the manuscript. All authors read and approved the final manuscript.
